# Setanaxib Added to Pembrolizumab for Fibroblast-Rich, Recurrent, or Metastatic Squamous Cell Carcinoma of the Head and Neck: A Phase II Randomized Study

**DOI:** 10.1158/2767-9764.CRC-26-0422

**Published:** 2026-10-21

**Authors:** Jérôme Fayette, Gareth J. Thomas, Amaury Daste, Maciej Rotarski, Beatriz Castelo, Antonio Rullan, Aaron Levine, Richard Philipson, Benjamin H. Jenkins, Kevin J. Harrington

**Affiliations:** 1 Medicine Department, Centre Léon Bérard, Lyon, France.; 2 School of Cancer Sciences, University of Southampton, Southampton, United Kingdom.; 3 Medical Oncology, CHU Bordeaux – Hôpital St. André, Bordeaux, France.; 4 Oncology Department, Centre Oncologie du Pays Basque, Bayonne, France.; 5 Medical Oncology, Hospital Universitario La Paz, Madrid, Spain.; 6 Head and Neck Unit, The Royal Marsden Hospital – Chelsea, London, United Kingdom.; 7 Statistics Department, Calliditas Therapeutics AB, Stockholm, Sweden.; 8 Clinical Department, Calliditas Therapeutics AB, Stockholm, Sweden.; 9 Division of Radiotherapy and Imaging, The Institute of Cancer Research, Royal Marsden, London, United Kingdom.

## Abstract

**Purpose:**

The efficacy of checkpoint inhibitors (e.g., pembrolizumab) in head and neck squamous cell carcinoma (HNSCC) is limited by cancer-associated fibroblasts (CAFs). This randomized, double-blind, placebo-controlled, phase II trial (NCT05323656) evaluated the efficacy and safety of the reactive oxygen species inhibitor setanaxib with pembrolizumab in patients with CAF-rich HNSCC.

**Patients and Methods:**

Participants (≥18 years) with recurrent or metastatic HNSCC with CAF tumor levels ≥5% received setanaxib (800 mg orally twice a day) or placebo for ≤105 weeks, plus intravenous pembrolizumab (200 mg every 3 weeks). The primary outcome was the best percentage change from baseline in tumor size. Secondary outcomes included progression-free survival (PFS), overall survival (OS), and changes in tumor levels of CAFs; cluster of differentiation 8 (CD8)^+^ tumor-infiltrating lymphocytes (TIL); and forkhead box P3 (FOXP3)^+^ regulatory T cells at week 9.

**Results:**

Fifty-five participants (setanaxib, *n* = 27; placebo, *n* = 28) were treated. Least squares (LS) mean (standard error) best percentage changes in tumor size from baseline values were −7.9% (9.3%; setanaxib) and −12.9% {9%; placebo; LS mean difference [80% confidence interval (CI)], 5.1 (−11.9 to 22); *P* = 0.7008}. PFS hazard ratio (HR; 80% CI) was 0.58 (0.38–0.89), favoring setanaxib [*P* = 0.1023 (threshold < 0.2)]. OS HR (80% CI) was 0.45 (0.24–0.85), also favoring setanaxib (*P* = 0.1057). Transcriptomic analyses identified a significant increase in CD8^+^ TILs with setanaxib (*P* = 0.037), but not placebo. There were 33 serious adverse events (16 setanaxib and 17 placebo), leading to four deaths (all placebo).

**Conclusions:**

These improvements in PFS, OS, and CD8^+^ TILs can guide further setanaxib research in patients with solid tumors.

**Significance:**

Early data suggest that for patients with cancers rich in fibroblasts, adding setanaxib to existing therapy could improve treatment response. This study suggests that combining pembrolizumab with setanaxib improved survival measures in patients with fibroblast-rich recurrent and/or metastatic head and neck cancer. These findings support further evaluation of setanaxib.

## Introduction

Myofibroblastic cancer–associated fibroblasts (myCAFs) are significantly associated with poor prognosis in head and neck squamous cell carcinoma (HNSCC), with around half of primary tumors, and an even higher proportion of recurrent or metastatic tumors, containing moderate or high myCAF levels (1). Conversion of normal fibroblasts into myCAFs is centrally regulated through TGFβ signaling. This process is, at least in part, triggered by an upregulation of NADPH oxygenase (NOX) 4, which catalyzes the formation of intracellular reactive oxygen species (ROS). NOX4 is widely expressed in cancers, including HNSCC, primarily in myCAFs (2). NOX4 has also been shown to play a pivotal role in regulating myofibroblast differentiation in various fibrotic diseases (3). In preclinical studies, inhibition of NOX4-mediated ROS production effectively prevented the differentiation of normal fibroblasts into myofibroblasts or myCAFs in both tumors and fibrosis (2, 4).

The effectiveness of anti–programmed cell death protein 1 (PD-1)/programmed cell death ligand 1 (PD-L1) immune checkpoint inhibitors is limited by factors that suppress the infiltration of cytotoxic T cells into tumors (5). myCAFs have emerged as a major mediator of T-cell exclusion from tumors; myCAF-rich tumors often have low levels of tumor-infiltrating lymphocytes (TIL) and, consequently, respond poorly to immunotherapy (6–10). Most solid malignancies, including HNSCC, contain myCAF-rich subgroups; the mesenchymal molecular subgroup of HNSCC is one such category (11).

In 2020, there were approximately 875,000 new cases of HNSCC worldwide, with around 450,000 deaths (12). Up to 50% of patients with locally advanced HNSCC treated with curative intent can be expected to experience disease recurrence or metastasis within 5 years (13). Pembrolizumab is now approved globally as a first-line treatment for recurrent and/or metastatic HNSCC, either as monotherapy (in PD-L1–positive disease) or in combination with platinum plus fluorouracil (independent of the PD-L1 status, in some jurisdictions; refs. 14, 15). Although this has significantly improved patients’ survival and response rates (16), more than 80% of patients fail to benefit (15).

Setanaxib is an orally available ROS inhibitor (17) that is well tolerated in healthy adults (18) and in adults with primary biliary cholangitis (19, 20). In preclinical cancer studies, setanaxib has been shown to overcome myCAF-mediated immunotherapy resistance, suppressing or reversing myCAF differentiation and significantly potentiating cluster of differentiation 8 (CD8)^+^ T-cell infiltration into tumors (8). These data suggest that for patients with myCAF-rich cancers, the addition of setanaxib to a PD-1 inhibitor–based regimen could potentially overcome myCAF-mediated CD8^+^ T-cell exclusion and, thereby, improve response and survival.

The goal of this trial (https://clinicaltrials.gov/study/NCT05323656; first submitted on April 5, 2022) was to evaluate the efficacy, safety, tolerability, and effects on tumor biomarkers of setanaxib in combination with the PD-1 inhibitor pembrolizumab in patients with myCAF-rich, recurrent, or metastatic HNSCC.

## Patients and Methods

### Study design

NCT05323656 was a hospital-based phase II, randomized, double-blind, placebo-controlled, proof-of-concept study (Supplementary Fig. S1) conducted in 18 centers across Europe (France, Germany, Italy, Poland, Spain, and the United Kingdom) and at one center in the United States, in accordance with the Declaration of Helsinki and the International Council for Harmonisation guidelines on Good Clinical Practice. The study protocol, patient recruitment procedures, and any information provided to patients were reviewed and approved by an independent review board or independent ethics committee at each participating center. Written informed consent was provided for all patients prior to their participation and could be withdrawn at any time; a separate consent was sought for the collection of an optional tumor biopsy at the time of disease progression.

### Participants

The study included individuals aged ≥18 years with recurrent or metastatic HNSCC. These were patients with a primary tumor of the oral cavity, nasal cavity, paranasal sinuses, oropharynx, hypopharynx, or larynx, confirmed histologically or cytologically, with or without nodal involvement or metastatic spread, and irrespective of human papillomavirus (HPV) status. Inclusion of patients with nasal/paranasal sinus cancers reflected a deliberate decision to include these tumor sites, recognizing that they present a significant unmet medical need but are frequently excluded from similar studies. Such patients frequently receive standard-of-care therapies, as defined by guidelines for patients with oral cavity, oropharynx, larynx, and hypopharynx cancers, in real-world clinical practice. A table summarizing the representativeness of the trial population is included (Supplementary Table S1).

Patients were included if they were eligible for first-line treatment for recurrent or metastatic HNSCC with pembrolizumab, as determined by the investigator. In addition, each participant was required to have tumor levels of CAFs ≥5% (a feature of poor prognosis as described previously; ref. 1), as assessed by a central laboratory on samples taken within 30 days prior to screening or on suitable archival tissue acquired within the previous 6 months, with no intervening anticancer therapy during that time. Other inclusion criteria included a combined positive score (CPS) ≥1, a life expectancy of at least 6 months according to the investigator, Eastern Cooperative Oncology Group (ECOG) performance status 0 or 1, and adequate organ and bone marrow function within 35 days of starting treatment, as measured via neutrophil, platelet, hemoglobin, and bilirubin levels; aspartate aminotransferase and alanine aminotransferase activity; serum creatinine levels; or creatinine clearance.

Patients were excluded if they had a tumor eligible for curative-intent surgical resection, immunosuppressive therapy within 7 days, anticancer mAb treatment within 4 weeks, chemotherapy or targeted small-molecule therapy, or radiotherapy within 2 weeks of the first dose of study therapy (ensuring that all patients were treatment-naïve for relapsed/metastatic disease). Other exclusion criteria included unresolved adverse events (AEs) of grade 2 or greater (except alopecia) due to previous therapy, treatment with an investigational agent within 12 weeks of the screening visit, central nervous system metastases or carcinomatous meningitis, known additional malignancy that was progressing or required active treatment (except basal cell carcinoma of the skin, squamous cell carcinoma of the skin, or *in situ* cervical cancer that had undergone therapy) or treated malignancy with no known active disease within 2 years of the first dose and which had a low potential risk for recurrence, or conditions that constituted a risk or contraindication to patient participation or the validity of results (e.g., active autoimmune disease requiring systemic treatment, active infection requiring systemic therapy, or unstable cardiovascular disease).

Recruitment was conducted via regular clinical service at participating centers. Sex or gender was reported by the investigator.

### Randomization and masking

Randomization (1:1) was stratified by HPV status and performed via centralized interactive response technology. Where the HPV status was not known or not documented in the medical records, HPV was determined before randomization as per site standard operating procedures [e.g., p16 IHC or HPV DNA detection (PCR or ISH)]. The testing methodology was recorded in the electronic case report form. Each participant received a unique randomization code generated by the randomization statistician, who stored the master randomization list in a secure system. The randomization statistician was not involved in the rest of the trial. The investigator, site personnel, sponsor, and sponsor’s representatives involved in monitoring and conducting the study, as well as the participants, were all blinded to treatment assignments. Blinding was achieved via visually identical setanaxib and placebo tablets, which were kept in bottles that were coded and labeled in a manner that protected blinding.

Breaking of the blind was only allowed in an emergency, at the discretion of the investigator, following a prespecified procedure. In addition, an unblinded statistician and programmers were responsible for providing safety data to an unblinded independent data monitoring committee, which performed its first assessment of safety and tolerability after 12 patients had received at least one cycle of pembrolizumab and, thereafter, periodically as needed.

### Procedures

Participants received setanaxib (Calliditas Therapeutics) 800 mg twice daily orally (or via feeding tube or a percutaneous endoscopic gastrostomy device) or matching placebo for up to 105 weeks (2 years), as well as pembrolizumab (KEYTRUDA, Merck) 200 mg, infused intravenously every 3 weeks. After the last study dose, participants were followed for safety for an additional 28 days. Treatment was stopped if a patient experienced unacceptable toxicity or radiologically confirmed disease progression. Tumor size was determined via CT or MRI at screening, week 9, then every 6 weeks in the first year, and then every 9 weeks until the end of treatment. Tumor biopsies were taken during screening or within the preceding 30 days and again at 9 weeks after the start of study treatment (or sooner, in case of disease progression).

### Outcomes

The primary outcome was the best percentage change from baseline in tumor size, as measured by the sum of diameters of target lesions, per Response Evaluation Criteria in Solid Tumors version 1.1 (RECIST v1.1) criteria (21, 22). Key secondary outcomes were as follows: progression-free survival (PFS; defined as the time from randomization to the first documented disease progression or death due to any cause) at 3, 6, and 12 months; median PFS; and changes from baseline to week 9 in the levels of tumor tissue CAFs, CD8^+^ TILs, and regulatory T cells (Treg). Other secondary endpoints were as follows: overall response rate [ORR; defined as the proportion of patients who achieved a complete response (CR) or partial response (PR)]; duration of response (DoR; defined as the minimum time from when CR or PR was first observed to the time of disease progression or death); disease control rate (DCR; defined as the proportion of patients in whom the best overall response was determined as CR, PR, or stable disease); overall survival (OS; defined as the time from randomization to death due to any cause, in patients who did not experience a censoring event by the last follow-up); expression levels of PD-L1 in tumor tissue using the PD-L1 IHC 22C3 pharmDx assay (Agilent Technologies; www.agilent.com; code GE006); expression of genes associated with gene activation for CAFs, CD8^+^ TILs, and Tregs, as measured by ribonucleic acid (RNA) sequencing (RNA-seq); and pre- and postdose plasma concentrations of setanaxib and its metabolite GKT138184. Tumor size, disease progression events, PFS, ORR, DoR, DCR, CR, PR, and stable disease were defined based on the RECIST v1.1 (21, 22).

Exploratory endpoints included the relationship between the pharmacokinetics and pharmacodynamics of setanaxib and GKT138184; expression of immune cell and stromal cell gene signatures, as measured by RNA-seq (see “Transcriptome analysis”); and the circulating levels of biomarkers in peripheral blood, including cytokines, chemokines, T-lymphocyte receptor repertoires, and immunomodulation-associated gene expression biomarkers. For the pharmacokinetics analysis, individual patient exposures were estimated using a prior phase I healthy subject population pharmacokinetic model; this was done to verify that the exposures in the current study were as expected.

Safety and tolerability outcomes included AEs, with anemia and hypothyroidism designated as AEs of special interest (AESI); vital signs; clinical laboratory variables; and physical examination findings.

### IHC

Double immunostaining for CD8 and forkhead box P3 (FOXP3) T cells was performed and analyzed as previously described (23). Briefly, intratumoral CD8^+^ and FOXP3^+^ TILs were counted in multiple random high-power fields (HPF) at 400× magnification. Where possible, 10 HPF were assessed. CAFs were identified centrally in formalin-fixed, paraffin-embedded (FFPE) tissue sections using IHC detection of α-smooth muscle actin, as described previously (1). A semiquantitative scoring system based on CAF stroma positivity was used to classify tumors into three groups (low, <5%; moderate, from 5% to 50%; and high, >50%). PD-L1 expression was assessed using CPS (22CS).

### Transcriptome analysis

Transcriptome analysis of pre- and on-treatment FFPE samples was performed using the HTG EdgeSeq Panel (19,398 genes; HTG Molecular Diagnostics) with single-read deep sequencing performed on an Illumina NextSeq 500 (Illumina, Inc.; RRID: SCR_014983). Minimum input was an 11-mm^2^ area from a 5 μm FFPE section. Raw read counts were converted to counts per million (CPM) using the edgeR package (v3.40.2; Bioconductor open-source software; https://bioconductor.org/packages/edgeR; RRID: SCR_012802). CPM-normalized counts were uploaded to CIBERSORTx (Stanford University; https://cibersortx.stanford.edu/; RRID: SCR_016955) as mixture files (24). Cell type fractions were deconvoluted using the validated leukocyte gene signature matrix, LM22, as published previously (25). Cell fraction imputation was performed using bulk mode batch correction and run in absolute mode with 100 permutations. For enumeration of myCAFs using CIBERSORTx, a validated custom signature matrix was implemented, uploading annotated HNSCC single-cell RNA-seq data (10x Chromium) as a single-cell reference matrix, as published previously (26). The signature matrix was created with the minimum expression set at 0.05 and the number of barcode genes set at between 100 and 500 (all other parameters were set to default). In this case, cell fraction imputation was performed using single-cell mode batch correction, in absolute mode with 100 permutations.

### Statistical analysis

For the primary outcome, the overall sample size of 50 patients was determined to provide 85% power to detect a 20% mean difference between treatment groups, with an estimated standard deviation (SD) of 30% and a two-sided alpha of 0.2. The key secondary outcomes of the numbers of CAFs, CD8^+^ TILs, and Tregs in tumor tissue and gene expression analysis in tumor tissue were used to determine proof of concept. With 25 patients per treatment group, there would be 90% power to detect a limit fold change of more than 1.5 in gene-sequencing endpoints measured in tumor tissue, assuming at least 20 patients had an evaluable baseline and postbaseline tissue sample. For the key secondary outcome of PFS, with a true PFS hazard ratio (HR) of 0.5, 38 progression events would be needed to yield approximately 80% power to detect a difference in PFS with a two-sided alpha of 0.2.

The primary efficacy outcome was analyzed based on the full analysis set (FAS; based on treatment allocation, irrespective of the actual treatment received), using an analysis of covariance (ANCOVA) model with treatment and baseline tumor size as covariates. Postbaseline levels of CAFs, CD8^+^ TILs, and Tregs in tumor tissue, as well as the PD-L1 expression levels, were analyzed using an ANCOVA model with treatment as a fixed factor and with baseline values as covariates (two-sided alpha = 0.2).

PFS was summarized using the Kaplan–Meier method, with median estimates and 80% confidence intervals (CI) provided at 3, 6, and 12 months. Patients who had not progressed by the time of the final cutoff were censored. Between-group comparisons were conducted using a fitted Cox proportional hazards model with treatment group as the only covariate. HR was based on the profile partial likelihood from the Cox model and calculated as a measure of the treatment effect, along with the Wald 80% CIs and the asymptotic *P* value from the Wald test for the difference in the log hazards.

OS and DoR data were analyzed using Kaplan–Meier methodology. ORR and DCR data were summarized descriptively, with Agresti–Coull 90% CIs. Changes in patterns of gene expression and differential gene expression were analyzed using principal component analysis. In transcriptomic analyses, abundance comparisons between different cell fractions at baseline and within individual cell fractions between baseline and week 9 were performed using the Wilcoxon signed-rank test (two-sided alpha = 0.05).

Safety analyses were based on the safety analysis set, which included all participants who received at least one tablet of setanaxib or placebo (analyzed according to the treatment they actually received, irrespective of treatment allocation) and were presented descriptively.

## Results

The first patient was treated on May 13, 2022, and the last patient’s last treatment was received on October 25, 2023. Of 118 screened individuals, 55 were randomized (setanaxib, *n* = 27; placebo, *n* = 28; Supplementary Fig. S2). The most common reasons for screen failure were CAF level in tumors <5% (*n* = 31), CPS < 1 (*n* = 10), and thyroid-stimulating hormone (TSH) > upper limit of normal at screening (*n* = 5). All randomized patients were included in the FAS, per-protocol analysis set, and safety analysis set, and all patients randomized to receive setanaxib were included in the pharmacokinetics analysis set. All participants received treatment as allocated.

Twenty (74%) patients in the setanaxib arm and 21 (75%) patients in the placebo arm discontinued treatment, predominantly because of progressive disease [setanaxib, *n* = 15 (55%); placebo, *n* = 14 (50%); Supplementary Fig. S2]. No patient was lost to follow-up. Overall, the median (IQR) age was 65 (60–71) years, the mean (SD) body mass index was 23.4 (4.3) kg/m^2^, and the majority of patients were White [38/55 (69%)]. Demographic and clinical characteristics at baseline were generally well balanced between treatment groups, with a few exceptions. Compared with the setanaxib group, the placebo group had a smaller proportion of men [64% (18/28) vs. 81% (22/27)] and patients with an ECOG score of 0 [29% (8/28) vs. 63% (17/27)], a greater proportion of individuals with T4 stage primary tumors [17/28 (61%) vs. 10/27 (37%)] or distant metastases [5/28 (18%) vs. 1/27 (4%)], and a shorter mean (SD) time since diagnosis [21 (27) months vs. 32 (42) months; Table 1].

**Table 1.** Baseline characteristics.

Characteristic	Setanaxib 800 mg twice daily + pembrolizumab 200 mg every 3 weeks (*n* = 27)	Placebo + pembrolizumab 200 mg every 3 weeks (*n* = 28)	Overall (*N* = 55)
Age, years, median (IQR)	65 (60–71)	65 (59.5–69.5)	65 (60–71)
Sex, *n* (%)	​	​	​
Male	22 (81.5)	18 (64.3)	40 (72.7)
Female	5 (18.5)	10 (35.7)	15 (27.3)
Race, *n* (%)	​	​	​
Asian	1 (3.7)	0	1 (1.8)
White	16 (59.3)	22 (78.6)	38 (69.1)
Not reported or unknown	10 (37)	6 (21.4)	16 (29.1)
BMI, kg/m^2^, mean (SD)	24.4 (4.6)	22.4 (3.9)	23.4 (4.3)
ECOG grade, *n* (%)^a^	​	​	​
0	17 (63)	8 (28.6)	25 (45.5)
1	10 (37)	20 (71.4)	30 (54.5)
≥2	0	0	0
HPV status, *n* (%)	​	​	​
Positive	5 (18.5)	5 (17.9)	10 (18.2)
Negative	22 (81.5)	23 (82.1)	45 (81.8)
Actual PD-L1 values, CPS, mean (SD)	19.7 (29.4)	13.1 (25.1)	17.2 (27.3)
Time since diagnosis, months, mean (SD)	32.4 (42)	20.7 (27)	26.7 (35.6)
Tumor location, *n* (%)	​	​	​
Hypopharynx	1 (3.7)	1 (3.6)	2 (3.6)
Larynx	6 (22.2)	2 (7.1)	8 (14.5)
Nasal cavity	0	1 (3.6)	1 (1.8)
Oral cavity	6 (22.2)	11 (39.3)	17 (30.9)
Oropharynx	10 (37)	5 (17.9)	15 (27.3)
Paranasal sinus	1 (3.7)	0	1 (1.8)
Parotid gland	0	1 (3.6)	1 (1.8)
Tongue	0	2 (7.1)	2 (3.6)
Tonsil	2 (7.4)	0	2 (3.6)
Other	1 (3.7)	5 (17.9)	6 (10.9)
Primary tumor (T) stage, *n* (%)	27 (100)	27 (96.4)	54 (98.2)
T1	3 (11.1)	1 (3.6)	4 (7.3)
T2	5 (18.5)	5 (17.9)	10 (18.2)
T3	9 (33.3)	4 (14.3)	13 (23.6)
T4	6 (22.2)	13 (46.4)	19 (34.5)
T4a	2 (7.4)	4 (14.3)	6 (10.9)
T4b	2 (7.4)	0	2 (3.6)
Regional lymph nodes (N) stage	27 (100)	27 (96.4)	54 (98.2)
N0	5 (18.5)	7 (25)	12 (21.8)
N1	2 (7.4)	2 (7.1)	4 (7.3)
N2	4 (14.8)	4 (14.3)	8 (14.5)
N2a	1 (3.7)	1 (3.6)	2 (3.6)
N2b	3 (11.1)	7 (25)	10 (18.2)
N2c	8 (29.6)	2 (7.1)	10 (18.2)
N3	1 (3.7)	1 (3.6)	2 (3.6)
N3b	3 (11.1)	3 (10.7)	6 (10.9)
Distant metastasis (M) stage	27 (100)	27 (96.4)	54 (98.2)
M0	26 (96.3)	22 (78.6)	48 (87.3)
M1	1 (3.7)	5 (17.9)	6 (10.9)
Any prior chemotherapy received by >15% of patients, *n* (%)	​	​	​
Cisplatin	16 (59.3)	15 (53.6)	31 (56.4)
Fluorouracil	6 (22.2)	6 (21.4)	12 (21.8)
Docetaxel	4 (14.8)	6 (21.4)	10 (18.2)

Abbreviation: BMI, body mass index.

a ECOG grade 0 = fully active and able to do all predisease activities with some limitations; 1 = physically able to move around and do light or sedentary work but cannot do anything too strenuous; 2 = able to move around and care for themselves, but they cannot do any work; 3 = not able to do much for themselves and have to stay in bed or a chair more than half the time they are awake; and 4 = completely unable to take care of themselves and stuck in bed or a chair.

The most common medical history findings by system organ class were surgical and medical procedures (60%), vascular disorders (56%), and gastrointestinal disorders (44%). Prior anticancer medication use was generally consistent between groups (Supplementary Table S2). Twenty-four (44%) patients had received one previous radiotherapy, and 22 (40%) had received two or more. In total, 26 (47%) patients had prior cancer surgery.

The mean (SD) duration of exposure was 20 (14) weeks for the setanaxib group and 16 (15) weeks for the placebo group. Twenty (74%) and nineteen (68%) patients in the setanaxib and placebo groups, respectively, missed at least one dose of setanaxib or placebo, with AEs as the primary reason in 12 (44%) and 7 (25%) patients. Dose reductions were reported in two patients receiving setanaxib, both due to reported AEs.

**Figure 1.**
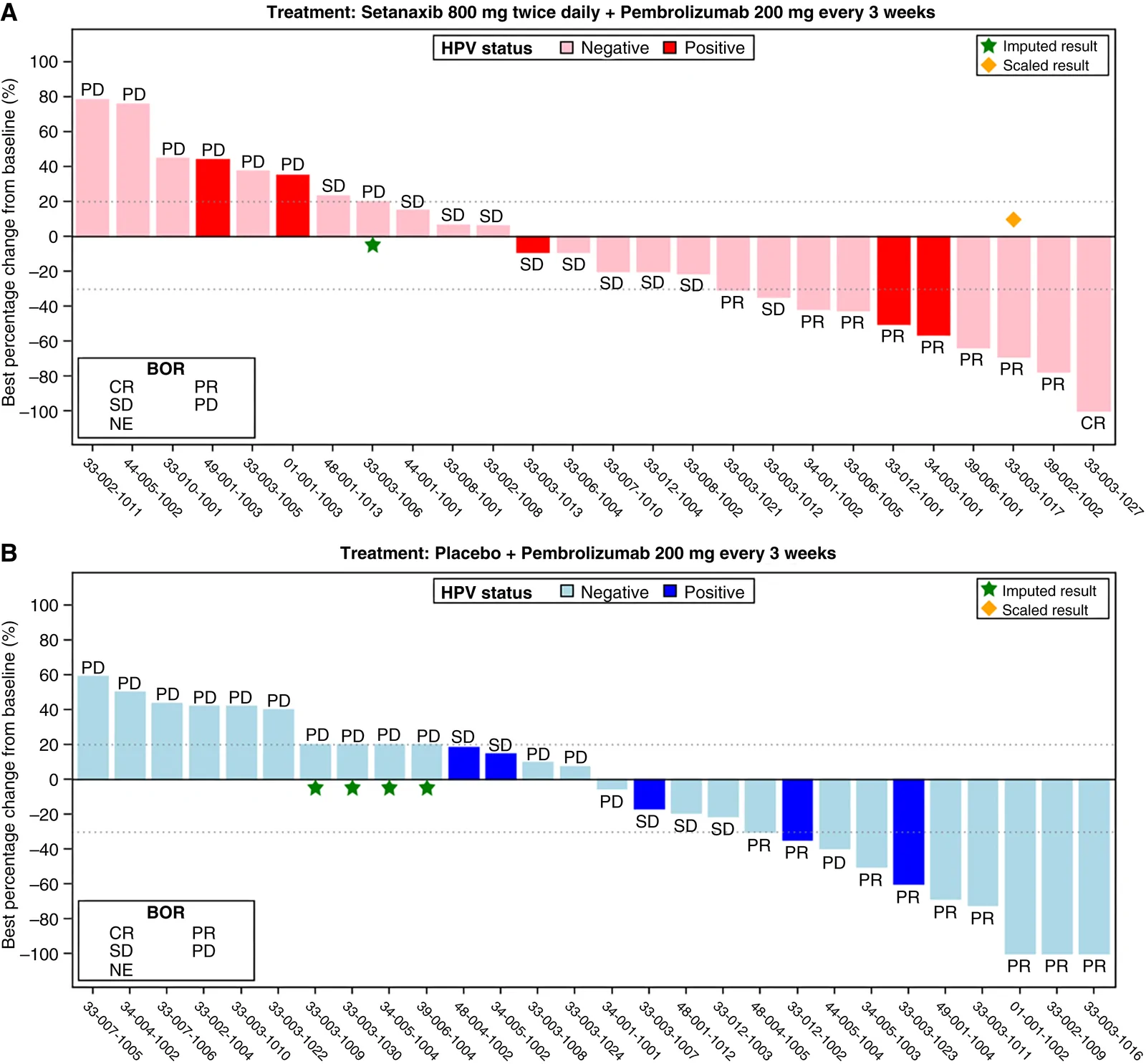
Waterfall plot of best percentage change from baseline in tumor size with (**A**) setanaxib + pembrolizumab and (**B**) placebo + pembrolizumab. Baseline is defined as the last nonmissing value before the first dose of treatment. Tumor size was defined as the sum of the longest diameters of the target lesions based on RECIST assessments, and the best percentage change from baseline in tumor size was derived as the largest percentage decrease or the smallest percentage increase in tumor size from baseline. BOR, best overall response; NE, not evaluable; PD, progressive disease; SD, stable disease.

For the primary endpoint of best percentage change in tumor size from baseline, the least squares (LS) mean [standard error (SE)] values were −7.9% (9.3%) for the setanaxib group and −12.9% (9%) for the placebo group [LS mean difference (80% CI), 5.1 (−11.9 to 22); *P* = 0.7008; Fig. 1A and B]. Disease progression or death was observed in most patients in both groups [setanaxib, *n* = 17 (63%); placebo, *n* = 21 (75%)]. Ten (37%) and seven (25%) patients in the setanaxib and placebo groups were censored, respectively, of whom nine (33%) and five (18%) were alive and progression-free (Supplementary Table S3).

The PFS HR (80% CI) was 0.58 (0.38–0.89) in favor of setanaxib [*P* = 0.1023 (predefined threshold: *P* < 0.2)]. In a *post hoc* sensitivity analysis, exclusion of patients with distant metastases [the largest relative percentage discrepancy at baseline, 4% (setanaxib) vs. 18% (placebo)] resulted in a similar PFS [HR (80% CI), 0.61 (0.39–0.95)]. An additional *post hoc* sensitivity analysis in which PFS was adjusted for baseline ECOG scores also resulted in a similar HR for PFS (Supplementary Fig. S3A and S3B).

**Figure 2.**
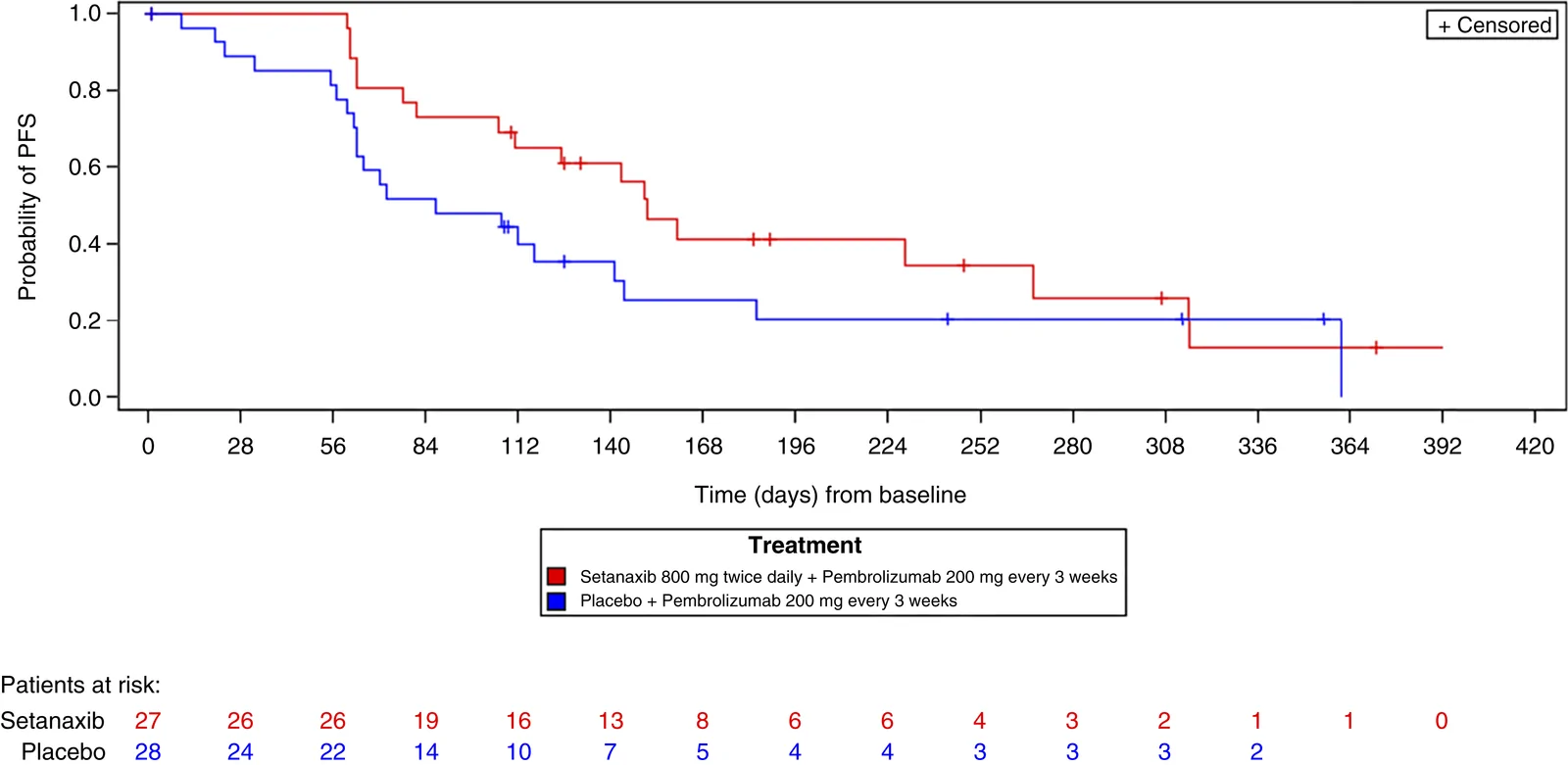
Kaplan–Meier plot of PFS, FAS. PFS time (days) was calculated as follows: [(date of disease progression or censoring) – (date of randomization)] + 1. Patients who were not observed to have encountered the event were censored based on the rules detailed in the statistical analysis plan. Overall visit responses were programmatically derived per RECIST v1.1, using the target response, nontarget response, and new lesion indicator, collected on the Disease Response page of the electronic case report form for each visit.

The median PFS was 151 days in the setanaxib group and 87 days in the placebo group (Fig. 2). At 3 months, the proportions (80% CI) of patients in the setanaxib and placebo groups who survived progression-free were 73% (62%–84%) and 48% (36%–61%), respectively; those values were 41% (28%–55%) and 25% (14%–37%) at 6 months and 13% (0%–27%) and 0% at 12 months.

Among patients receiving setanaxib, 1 (4%) had a CR, 8 (30%) had a PR, 10 (37%) had stable disease, and 7 (26%) had progression of disease; among patients receiving placebo, none achieved CR, 9 (32%) had a PR, 5 (18%) had stable disease, and 14 (50%) had progression of disease (Fig. 2). ORR (90% CI) values were similar [setanaxib, 33% (20%–49%); placebo, 32% (21%–48%)], with nine patients in each experiencing a response. Disease control was achieved in 19 patients treated with setanaxib [DCR (90% CI), 70% (55%–83%)] and in 14 patients treated with placebo [50% (35%–65%)]. The median DoR was not reached in the setanaxib group; in the placebo group, the median DoR was 79 days (Supplementary Fig. S4). In the setanaxib group, 6 (22%) patients died due to any cause, and 21 (78%) were censored compared with 13 (46%) and 15 (54%) patients in the placebo group, respectively (Supplementary Table S4; Supplementary Fig. S5). The OS HR (80% CI) was 0.45 (0.24–0.85) in favor of setanaxib [*P* = 0.1057 (*post hoc* nominal value with predefined threshold: *P* < 0.2)]. In the setanaxib group, 96% (80% CI, 91%–100%), 92% (86%–99%), and 88% (79%–96%) of patients were alive at 3, 6, and 9 months, respectively. In the placebo group, those values were 79% (80% CI, 69%–89%), 68% (57%–79%), and 58% (46%–71%).

**Figure 3.**
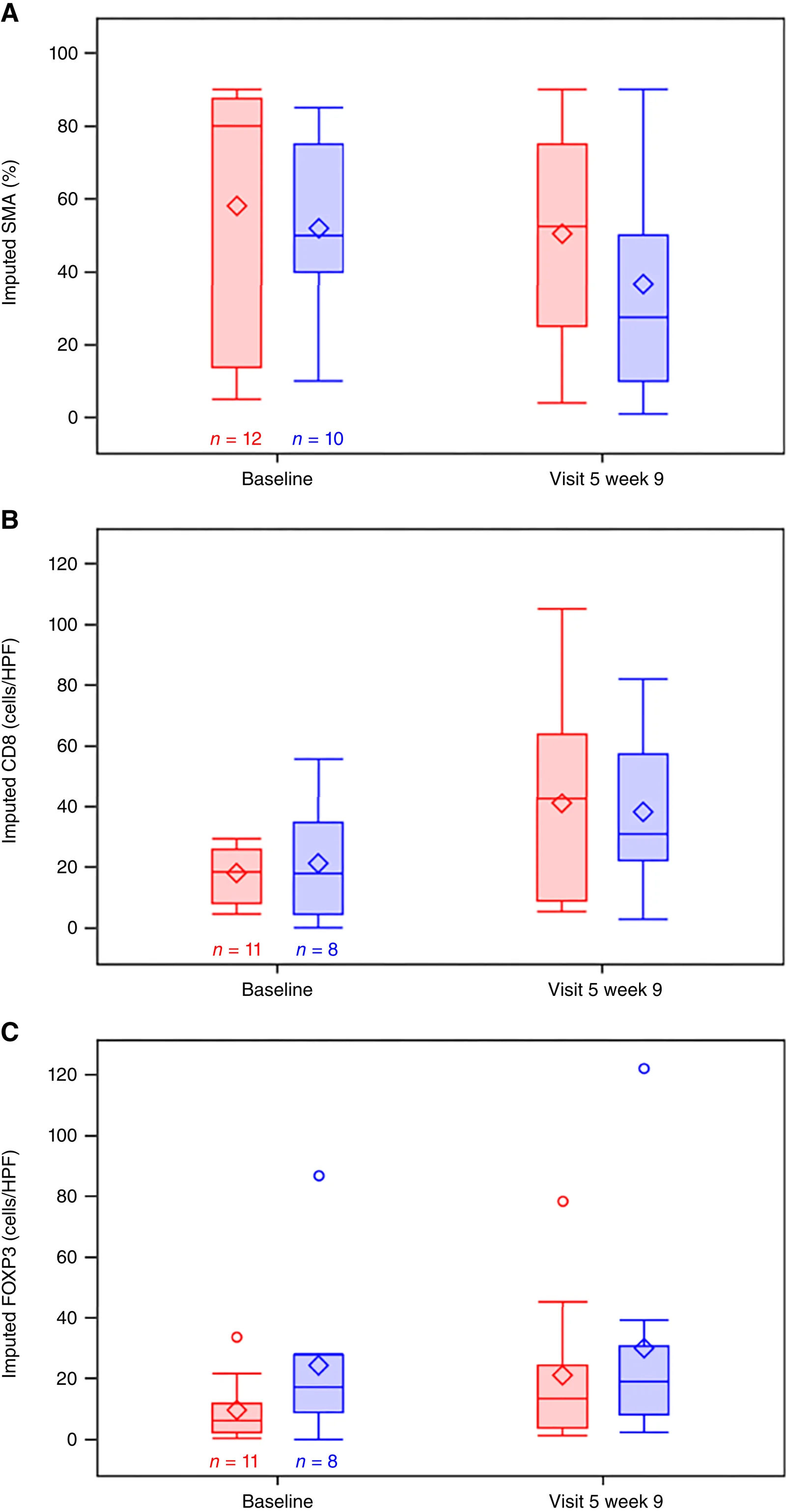
Box and whisker plot of the baseline and postbaseline levels of (**A**) CAFs [smooth muscle actin (SMA) %], (**B**) CD8^+^ TILs (cells/HPF), and (**C**) FOXP3^+^ Tregs (cells/HPF) by analysis visit (FAS). Red, setanaxib 800 mg twice daily + pembrolizumab 200 mg every 3 weeks; blue, pembrolizumab 200 mg every 3 weeks. Boxes are composed of quartile1 (Q1), median, and Q3. The diamond represents the group mean and the bars extending from the box mark the high/low whiskers (1.5× interquartile range above/below the Q3/Q1 values, respectively, drawn up to the largest/smallest observed data point within this distance). Outlier values within a group are plotted as separate points beyond the whiskers. Baseline was defined as the last nonmissing value before the first dose of investigational medicinal product. All visits (including unscheduled, follow-up visits, and end-of-treatment visits) were windowed to the appropriate analysis visit regardless of the visit label entered in the electronic case report form. Zero values were replaced with a constant equal to half of the smallest observed nonzero result across all patients. Only patients with both baseline and postbaseline values were included in this figure.

**Figure 4.**
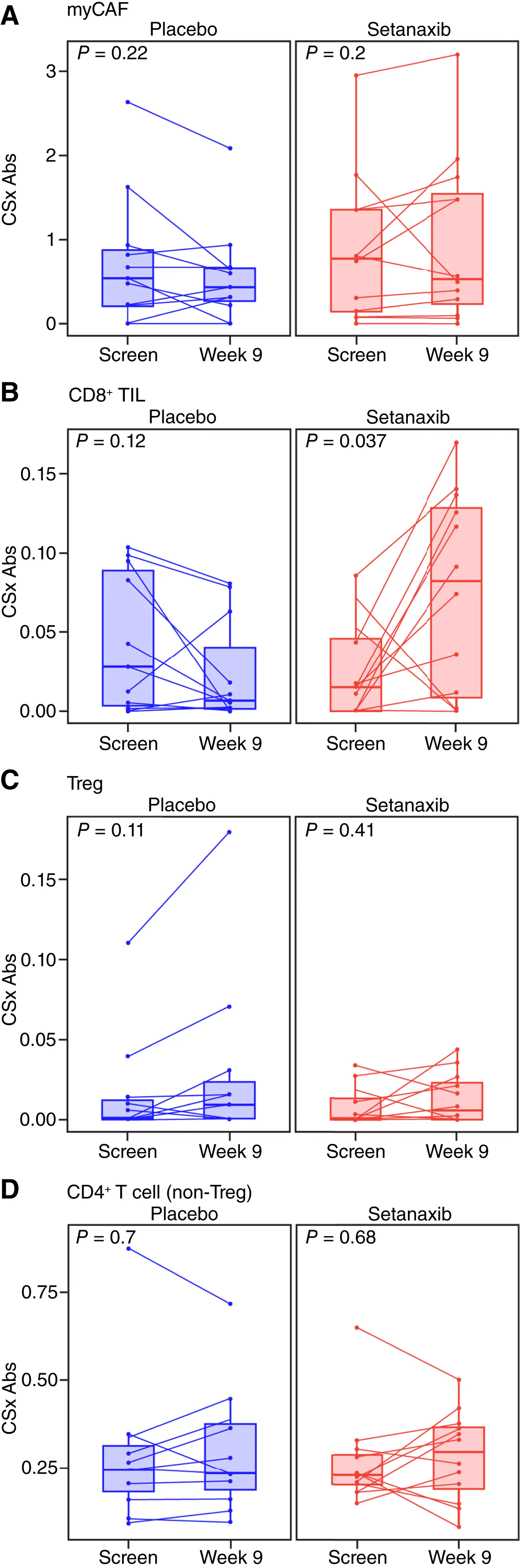
Transcriptome analysis—change in CIBERSORTx absolute abundance signature scores from screening to week 9 (on-treatment) for (**A**) myCAFs, (**B**) CD8^+^ TILs, (**C**) Tregs, and (**D**) CD4^+^ T cells (non-Treg). CSx Abs, CIBERSORTx absolute values.

From baseline to week 9, there was a mean (SD) decrease in CAF percentage in tumor tissue: from 58% (37) at baseline to 51% (31) with setanaxib and from 52% (23) to 37% (30) with placebo (Fig. 3). However, the LS mean differences (80% CI) between adjusted LS mean (SE) postbaseline CAF levels were 48% (6) with setanaxib and 39% (7) with placebo [9.5% (−2.3 to 21.3), *P* = 0.2986]. Similarly, applying CIBERSORTx deconvolution to gene expression data, there were no differences in myCAF abundance before and during treatment in either group (Fig. 4).

In the setanaxib group, microscopy analysis revealed a mean (SD) increase in CD8^+^ TIL levels from 18 (9) cells/HPF at baseline to 41 (35) cells/HPF at 9 weeks compared with an increase from 21 (20) cells/HPF to 38 (27) cells/HPF in the placebo group (Fig. 3), but the adjusted postbaseline LS mean (SE) levels were 43 (8) cells/HPF with setanaxib and 36 (9) cells/HPF with placebo (*P* = 0.59). The transcriptome analysis showed a significant increase in the number of CD8^+^ TILs from baseline to week 9 in the setanaxib group (*P* = 0.037), which was not observed in the placebo group (*P* = 0.12; Fig. 4). At an individual patient level, out of the 12 patients in the setanaxib group with available transcriptome data, 9 (75%) had an increase in the absolute number of CD8^+^ TILs in the tumor sample, and 7 of those 9 patients (78%) had a PR or stable disease. In the placebo group, 3 of 11 patients (27%) had an increase in CD8^+^ TILs in their tumor samples; of those, two had stable disease (Supplementary Fig. S6).

The mean (SD) levels of FOXP3^+^ Treg also increased in both treatment groups from baseline to week 9 [setanaxib: from 10 (10) cells/HPF to 21 (23) cells/HPF; placebo: from 24 (27) cells/HPF to 30 (39) cells/HPF; Fig. 3], but the adjusted postbaseline LS mean (SE) levels were 30 (5) cells/HPF with setanaxib and 19 (6) cells/HPF with placebo (*P* = 0.21). In the transcriptome analysis, no significant differences were observed in either Treg numbers or non-Treg CD4^+^ T-cell numbers (Fig. 4). In the setanaxib group, the mean (SD) PD-L1 levels increased from 20 (29) at baseline to 42 (38) at week 9; in the placebo group, the corresponding increase was from 13 (25) to 19 (16). The difference in adjusted postbaseline LS (SE) means was 40 (6) with setanaxib and 22 (7) with placebo (*P* = 0.078).

In a *post hoc* analysis of the entire study sample, there was a significant positive correlation (*P* = 0.0048) between the change in proportion of CD8^+^ TILs and the change in level of PD-L1 (*r*^2^ = 0.44; Supplementary Fig. S7).

Pharmacokinetic data were available for all 27 patients in the setanaxib group. Most pharmacokinetic samples were taken between 12 and 18 hours after the dose, and the observed plasma concentrations for setanaxib and GKT138184 were as expected based on existing phase I data in healthy subjects. Similar exposures to setanaxib and GKT138184 were estimated for when setanaxib was administered as a suspension via a percutaneous endoscopic gastrostomy device (*n* = 2) or as a tablet.

There were no substantial differences in the occurrence of treatment-emergent AEs (TEAE) between treatment groups. TEAEs occurred in all 27 patients receiving setanaxib and in 26 (93%) patients receiving placebo (Table 2); of these, 15 (56%) and 9 (32%) individuals, respectively, experienced TEAEs considered related to treatment. The most common TEAEs overall were asthenia [36% (20/55)], diarrhea [22% (12/55)], dyspnea [22% (12/55)], nausea [22% (12/55)], pruritus [22% (12/55)], and hypothyroidism [20% (11/55); Table 2]. TEAE occurrence was largely similar between treatment groups, but hypothyroidism was more common with setanaxib [30% (8/27)] than with placebo [11% (3/28)]. Hypothyroidism events with TSH ≥10 mIU/L were considered AESIs, which were observed in 4 of 27 (15%) of setanaxib-treated patients (5 events in total) and in 2 of 28 (7%) of patients treated with placebo (3 events in total). All the hypothyroidism AESIs were considered related to pembrolizumab (with or without relationship to setanaxib or placebo), except for one patient in the setanaxib group, who had preexisting hypothyroidism that worsened during treatment. Additionally, three patients receiving placebo experienced a total of three grade ≥3 events of anemia (one of which was considered related to study treatment), and one patient receiving setanaxib experienced two AESIs of increased blood TSH (neither of which was considered related to study treatment).

**Table 2.** Summary of TEAEs.

​Category	Setanaxib 800 mg twice daily + pembrolizumab 200 mg every 3 weeks (*n* = 27)	Placebo + pembrolizumab 200 mg every 3 weeks (*n* = 28)	Overall (*N* = 55)
Any TEAE	27 (100)	26 (92.9)	53 (96.4)
Any severe TEAE (grade ≥3)	11 (40.7)	14 (50)	25 (45.5)
Any TEAE with a fatal outcome	0	4 (14.3)	4 (7.3)
Any serious TEAE	8 (29.6)	10 (35.7)	18 (32.7)
Any TEAE leading to discontinuation of IMP	6 (22.2)	1 (3.6)	7 (12.7)
Any TEAE leading to discontinuation of study	1 (3.7)	4 (14.3)	5 (9.1)
Any TEAE of special interest	8 (29.6)	3 (10.7)	11 (20)
Most common TEAEs (≥12% in overall population)			
Asthenia	11 (40.7)	9 (32.1)	20 (36.4)
Diarrhea	7 (25.9)	5 (17.9)	12 (21.8)
Dyspnea	7 (25.9)	5 (17.9)	12 (21.8)
Nausea	7 (25.9)	5 (17.9)	12 (21.8)
Pruritus	7 (25.9)	5 (17.9)	12 (21.8)
Hypothyroidism	8 (29.6)	3 (10.7)	11 (20)
Decreased appetite	4 (14.8)	6 (21.4)	10 (18.2)
Neck pain	4 (14.8)	4 (14.3)	8 (14.5)
Anemia	3 (11.1)	4 (14.3)	7 (12.7)
Arthralgia	3 (11.1)	4 (14.3)	7 (12.7)
Constipation	2 (7.4)	5 (17.9)	7 (12.7)
Vomiting	4 (14.8)	3 (10.7)	7 (12.7)

NOTE: Data are *n* (%).

Abbreviation: IMP, investigational medicinal product.

Eight (30%) patients in the setanaxib arm and 10 (36%) patients in the placebo arm experienced a total of 33 serious TEAEs (setanaxib: 16 events; placebo: 17 events). One serious TEAE in each treatment arm was considered related to treatment: increased troponin in a patient receiving setanaxib and anemia in a patient receiving placebo. Four (14%) patients died because of a serious TEAE, all in the placebo group (aspiration, *n* = 2; respiratory tract infection, *n* = 1; and respiratory failure, *n* = 1); none of those TEAEs were considered related to study treatment (Table 2). Severe (grade ≥3) TEAEs were experienced by 11 (41%) patients in the setanaxib group and 14 (50%) patients in the placebo group (Supplementary Table S5).

More patients experienced TEAEs that led to discontinuation of setanaxib than placebo [6 (22%) vs. 1 (4%); Table 2; Supplementary Table S6]; in the majority of these patients, the TEAEs leading to discontinuation were considered unrelated to treatment. Of the six patients who discontinued setanaxib, four continued on pembrolizumab; the two patients who also discontinued pembrolizumab did so because of gastrointestinal complaints. Conversely, TEAEs that led to discontinuation from the study overall were more frequent in the placebo group than in the setanaxib group [4 (14%) vs. 1 (4%)]. The single event leading to study discontinuation in the setanaxib group was colitis, which was considered related to pembrolizumab.

Overall, no clinically meaningful changes in hematologic parameters, serum chemistry parameters, or electrocardiograms were observed between treatment groups.

## Discussion

In animal models, setanaxib was shown to suppress intratumoral myCAF accumulation and tumor growth, to promote the influx of CD8^+^ TILs into tumors (8), and to overcome myCAF-mediated resistance to both anticancer vaccination and anti–PD-1 immunotherapies (8). This proof-of-concept clinical trial was designed to investigate the effects of setanaxib on solid tumors in patients with HNSCC because this patient population presents significant clinical challenges, with more than four fifths of patients not benefiting from pembrolizumab treatment. In addition, HNSCC provides the opportunity to collect biopsies for the serial evaluation of biomarkers, such as transcriptomics.

We opted for a restricted sample size and carefully adjusted false-positive and false-negative error rates, in keeping with the proposed methodology for “phase II screening trials” (27). Due to the restricted sample size, a hard clinical outcome such as PFS or OS was not chosen as the primary endpoint, with the assumption that the event rates would not provide sufficient statistical power. Therefore, the surrogate outcome of the best percentage change in tumor size was selected as the primary endpoint.

There were noted differences between treatment groups at baseline. However, PFS *post hoc* sensitivity analyses, which excluded patients with distant metastases (representing the largest relative baseline discrepancy) and adjusted for ECOG score, suggest that they did not affect efficacy or safety outcomes.

The primary endpoint was not substantially different between groups. This may have been influenced by the fact that in line with the RECIST-defined minimum threshold for progressive disease (21), an increase of at least 20% in tumor size was used when accounting for patients who died early without a follow-up scan. There were fewer early deaths (imputed results) in the setanaxib versus placebo group (1 vs. 4, respectively), which may explain the observed difference between OS and PFS. Had a different threshold been used (e.g., at least 60%, consistent with the changes observed in those patients who were scanned), the average reduction in tumor size in the placebo group would have been smaller. However, this is acknowledged as a limitation of this endpoint in a patient population with a poor prognosis overall.

Taking into account the restricted sample size, clinically relevant improvements in PFS and OS with setanaxib plus pembrolizumab versus placebo plus pembrolizumab were reported. Similar waterfall plots between the two study arms (Fig. 2) suggest that the PFS benefit was driven by the stabilization of disease. The difference in the number of patients who died due to serious TEAEs (*n* = 4, all of them in the placebo arm) was considered a potential driver of the OS benefit with setanaxib; however, a *post hoc* sensitivity analysis excluding these four patients reported an OS benefit with setanaxib that was directionally consistent with the main result (HR, 0.64; 80% CI, 0.32–1.26). In a *post hoc* analysis of KEYNOTE-048, a pembrolizumab trial in head and neck cancer, response to subsequent therapy was a major contributor to the OS benefits (15).

The placebo PFS data are consistent with published literature for pembrolizumab alone (KEYNOTE-040: 2.1 months; KEYNOTE-048: 3.2 months; refs. 28, 29), and the placebo OS data are consistent with the 9-month OS value of approximately 60% estimable from the published pembrolizumab Kaplan–Meier curve from KEYNOTE-048 (28). Six-month PFS is considered an appropriate primary endpoint for phase II trials of checkpoint inhibitors in patients with advanced solid cancers (30). As mentioned previously, however, the restricted sample size precluded its use as a primary endpoint in the current study.

There was also some evidence of more durable responses with setanaxib plus pembrolizumab versus placebo plus pembrolizumab. However, DoR in the placebo plus pembrolizumab arm was only 79 days compared with nearly 2 years with pembrolizumab alone in KEYNOTE-048 (28). This discrepancy is possibly due to the high CAF levels in the current study, which may have contributed to a generally worse outcome in the placebo plus pembrolizumab group.

There was no detectable effect on CD8^+^ TILs as evaluated using HPF microscopy. However, transcriptomic analysis suggested an increase in CD8^+^ TILs in the tumor tissue of patients treated with setanaxib, which was not observed in the placebo arm. This apparent discrepancy may be due to a higher sensitivity of transcriptomic analyses than HPF microscopy and needs to be confirmed in subsequent studies. If confirmed, this finding could be of considerable importance, as elevated intratumoral CD8^+^ TIL levels have been shown to predict response to therapy in patients with advanced HNSCC (31). Therefore, CD8^+^ TILs may emerge as a surrogate outcome measure in future trials.

The results of the *post hoc* analysis of the transcriptomics data suggest an association between levels of CD8^+^ TIL and PD-L1 expression, with higher levels of both in the setanaxib arm. This finding may suggest that PD-L1 expression is being driven by increased immune cell levels in the tumor, which is consistent with known regulatory mechanisms.

Levels of CD4^+^ T cells were not affected by setanaxib treatment, consistent with murine preclinical data (8).

In the phase I program, setanaxib has been generally well tolerated, with a low occurrence of AEs and no reported deaths, serious AEs, or severe AEs. Most TEAEs were mild and self-limiting and were considered not related to treatment by the investigators. In the current phase II trial, setanaxib plus pembrolizumab treatment was not associated with new safety signals and had an acceptable safety and tolerability profile in patients with CAF-enriched HNSCC.

Key limitations were those commonly observed with phase II studies: a small sample size and an absence of adjustments for multiple comparisons. The noted discrepancies between treatment arms in baseline demographics and clinical characteristics are likely the consequence of a small sample size. It was not feasible in this study to stratify patients by ECOG performance status, as fewer than 10 patients would be included in each stratum; patients were also already stratified by HPV status. However, a *post hoc* sensitivity analysis stratifying patients by ECOG performance status was subsequently completed. As mentioned above, the *post hoc* PFS analyses did not indicate that these discrepancies affected efficacy assessments. Eligibility was also based on CAF expression that could be determined using archival tissue in cases where fresh tumor biopsy tissue was not available. Due to the impact of radiation and hypoxia on CAF, the mandatory use of recent biopsy tissue from all patients would have been the optimal approach.

Although the results of this exploratory phase II study were not definitive, they point to the possibility that this combination of setanaxib and pembrolizumab could have utility in targeting CAF-enriched, “immune-excluded” tumors. These findings can be used as the basis for further investigations of the effects of setanaxib on solid tumors.

## Supplementary Material

Supplementary Figure S1 Study design

Supplementary Figure S2 Patient disposition

Supplementary Figure S3 Post hoc sensitivity analysis – progression-free survival adjusted for ECOG performance status at baseline; (A) ECOG score 0, (B) ECOG score 1

Supplementary Figure S4 Kaplan–Meier plot for duration of response

Supplementary Figure S5 Kaplan–Meier plot for overall survival

Supplementary Figure S6 Transcriptome analysis - absolute numbers of CD8+ TILs in tumor samples by patient and response

Supplementary Figure S7 Change in the proportion of CD8+ TILs versus change in PD-L1 levels (entire study sample)

Supplementary Table S1 Representativeness of trial participants

Supplementary Table S2 Prior, on-study, and subsequent therapies

Supplementary Table S3 Summary of progression-free survival

Supplementary Table S4 Summary of overall survival

Supplementary Table S5 Severe TEAEs (CTCAE grade ≥3) by system organ class (≥2 patients in either treatment group)

Supplementary Table S6 TEAEs leading to discontinuation of study drug by preferred term

## Data Availability

The data generated in this study are not publicly available due to the proprietary nature of the research but are available upon reasonable request. Data sharing requests should be submitted to medical.information@calliditas.com as a research proposal with an appropriate statistical analysis and dissemination plan.
