# Supplementary material for: Setanaxib Added to Pembrolizumab for Fibroblast-Rich, Recurrent, or Metastatic Squamous Cell Carcinoma of the Head and Neck: A Phase II Randomized Study
Source: Cancer Res Commun. 2026 Oct 21;6(10):2463–74. doi: 10.1158/2767-9764.CRC-26-0422 (PMC13684902; doi:10.1158/2767-9764.CRC-26-0422)
Supplement: Supplementary file 1 [file crc-26-0422_supplementary_figure_s1_suppsf1.docx]

**Supplementary Figure S1.** Study design

^a^For patients with a suitable archival tumor biopsy sample available, the archival sample may be used to assess CAF levels and determine eligibility (Note: this can only be performed after the patient has provided signed informed consent). This does not dispense with the need for a recent pre-study tumor biopsy.

^b^Or earlier if progression occurs before 9 weeks.

**Abbreviations:** BID, twice daily; CAF, cancer-associated fibroblast; IV, intravenous; PFS, progression-free survival; PO, orally (or via either a feeding tube or a percutaneous endoscopic gastrostomy device in case patients are unable to swallow tablets); Q3W, every 3 weeks; R, randomization; RECIST v1.1, Response Evaluation Criteria in Solid Tumors version 1.1.
