# Supplementary material for: Setanaxib Added to Pembrolizumab for Fibroblast-Rich, Recurrent, or Metastatic Squamous Cell Carcinoma of the Head and Neck: A Phase II Randomized Study
Source: Cancer Res Commun. 2026 Oct 21;6(10):2463–74. doi: 10.1158/2767-9764.CRC-26-0422 (PMC13684902; doi:10.1158/2767-9764.CRC-26-0422)
Supplement: Supplementary file 2 [file crc-26-0422_supplementary_figure_s2_suppsf2.docx]

**Supplementary Figure S2.** Patient disposition

- Not meeting criteria (*n* = 58)
  - CAFs level in tumors <5% (*n* = 31)
  - CPS <1 (*n* = 10)
  - TSH >ULN at screening (*n* = 5)
  - Patient not willing or able to provide informed consent (*n* = 2)
  - No measurable disease per RECIST (*n* = 2)
  - ECOG performance status >1 (*n* = 2)
  - Other condition (*n* = 2)
  - AST and ALT >3x ULN (*n* = 1)
  - Prior treatment with pembrolizumab (*n* = 1)
  - History of hematologic problems (*n* = 1)
  - QTcF >450 ms or >470 ms (*n* = 1)
- Withdrawal by patient (*n* = 2)
- Physician decision (*n* = 2)
- Dead (*n* = 1)

Signed informed consent (*n* = 118)

Randomized (*n* = 55)

Randomized to placebo (*n* = 28)

- Received allocated intervention
  (*n* = 28)
- Did not receive allocated intervention (*n* = 0)

Lost to follow-up (*n* = 0)

Discontinued from intervention^a^ (*n* = 21)

- Progressive disease (*n* = 14)
- Adverse event (*n* = 3)
- Dead (*n* = 2)
- Investigator decision (*n* = 1)
- Other (*n* = 1)

Patients included in all randomized set, FAS, PPS, and safety analysis set
(*n* = 28)

Excluded from analysis^b^ (*n* = 0)

Randomized to setanaxib (*n* = 27)

Lost to follow-up (*n* = 0)

Discontinued from intervention^a^ (*n* = 20)

- Progressive disease (*n* = 15)
- Adverse event (*n* = 2)
- Withdrawal by patient (*n* = 1)
- Dead (*n* = 1)
- Other (*n* = 1)

Patients included in all randomized set, FAS, PPS, safety analysis set, and PK analysis set (*n* = 27)

Enrollment

Allocation

Follow-up

Analysis

Not randomized (*n* = 63)

- Received allocated intervention
  (*n* = 27)
- Did not receive allocated intervention (*n* = 0)

^a^Reasons for discontinuation are based on the End-of-Treatment (IMP) eCRF page.
^b^No patient in the placebo group was included in the PK analysis set.

**Abbreviations:** ALT, alanine aminotransferase; AST, aspartate aminotransferase; CAF, cancer-associated fibroblast; CPS, combined positive score; ECOG, Eastern Cooperative Oncology Group; eCRF, electronic case report form; FAS, full analysis set; IMP, investigational medicinal product; PK, pharmacokinetic; PPS, per protocol set; QTcF, QT interval using Fridericia's formula; RECIST, Response Evaluation Criteria in Solid Tumors; TSH, thyroid-stimulating hormone; ULN, upper limit of normal.
