# Supplementary material for: Setanaxib Added to Pembrolizumab for Fibroblast-Rich, Recurrent, or Metastatic Squamous Cell Carcinoma of the Head and Neck: A Phase II Randomized Study
Source: Cancer Res Commun. 2026 Oct 21;6(10):2463–74. doi: 10.1158/2767-9764.CRC-26-0422 (PMC13684902; doi:10.1158/2767-9764.CRC-26-0422)
Supplement: Supplementary file 3 [file crc-26-0422_supplementary_figure_s3_suppsf3.docx]

**Supplementary Figure S3.** Post hoc sensitivity analysis^a^ **–** progression-free survival adjusted for ECOG performance status at baseline; (A) ECOG score 0, (B) ECOG score 1

A.

B.

^a^Data were censored at 180 days because of the small size of patient groups beyond this timepoint.

The HR (80% CI) for setanaxib vs placebo, holding the effect of ECOG score at baseline constant, was 0.68 (0.42, 1.09).

**Abbreviations:** CI, confidence interval; ECOG, Eastern Cooperative Oncology Group; HR, hazard ratio.
