# Supplementary material for: Setanaxib Added to Pembrolizumab for Fibroblast-Rich, Recurrent, or Metastatic Squamous Cell Carcinoma of the Head and Neck: A Phase II Randomized Study
Source: Cancer Res Commun. 2026 Oct 21;6(10):2463–74. doi: 10.1158/2767-9764.CRC-26-0422 (PMC13684902; doi:10.1158/2767-9764.CRC-26-0422)
Supplement: Supplementary file 5 [file crc-26-0422_supplementary_figure_s5_suppsf5.docx]

**Supplementary Figure S5.** Kaplan–Meier plot for overall survival

Overall survival (days) = (date of [death or censoring] – date of randomization) + 1.

Patients who were not observed to have encountered the event were censored based on the rules detailed in the statistical analysis plan.

**Abbreviations:** BID, twice daily; Q3W, every 3 weeks.
