# Supplementary material for: Setanaxib Added to Pembrolizumab for Fibroblast-Rich, Recurrent, or Metastatic Squamous Cell Carcinoma of the Head and Neck: A Phase II Randomized Study
Source: Cancer Res Commun. 2026 Oct 21;6(10):2463–74. doi: 10.1158/2767-9764.CRC-26-0422 (PMC13684902; doi:10.1158/2767-9764.CRC-26-0422)
Supplement: Supplementary file 6 [file crc-26-0422_supplementary_figure_s6_suppsf6.docx]

**Supplementary Figure S6.** Transcriptome analysis - absolute numbers of CD8+ TILs in tumor samples by patient and response

CIBERSORTx Absolute Abundance LM22 Signature Score for CD8+ TIL, using gene expression data.

**Abbreviations:** CD8, cluster of differentiation 8; PD, progressive disease; PR, partial response; SD, stable disease; TIL, tumor-infiltrating lymphocyte.
