# Supplementary material for: Setanaxib Added to Pembrolizumab for Fibroblast-Rich, Recurrent, or Metastatic Squamous Cell Carcinoma of the Head and Neck: A Phase II Randomized Study
Source: Cancer Res Commun. 2026 Oct 21;6(10):2463–74. doi: 10.1158/2767-9764.CRC-26-0422 (PMC13684902; doi:10.1158/2767-9764.CRC-26-0422)
Supplement: Supplementary file 7 [file crc-26-0422_supplementary_figure_s7_suppsf7.docx]

**Supplementary Figure S7.** Change in the proportion of CD8+ TILs versus change in PD-L1 levels (entire study sample)

**Abbreviations:** CD8, cluster of differentiation 8; CPS, combined positive score; PD-L1, programmed death-ligand 1; TIL, tumor-infiltrating lymphocyte.
