# Supplementary material for: Setanaxib Added to Pembrolizumab for Fibroblast-Rich, Recurrent, or Metastatic Squamous Cell Carcinoma of the Head and Neck: A Phase II Randomized Study
Source: Cancer Res Commun. 2026 Oct 21;6(10):2463–74. doi: 10.1158/2767-9764.CRC-26-0422 (PMC13684902; doi:10.1158/2767-9764.CRC-26-0422)
Supplement: Supplementary file 8 [file crc-26-0422_supplementary_table_s1_suppst1.docx]

**Supplementary Table S1.** Representativeness of trial participants

| **Cancer type(s)/subtype(s)/stage(s)/condition** | **HNSCC** |
| --- | --- |
| **Conditions related to:** |  |
| Sex | HNSCC is a predominantly male disease, reflecting known sex-related differences in risk factor exposure such as tobacco and alcohol use (1, 2). Head and neck cancer generally afflicts males 2× to 4× more often than females (1, 2). Males have a substantially higher risk than females for all forms of HNSCC, regardless of environmental or viral etiology (2). |
| Age | HNSCC is primarily a disease of older adults. Non-virally associated HNSCC typically presents around 66 years of age, whereas HPV-associated oropharyngeal cancer occurs at a younger median age of approximately 53 years (2). |
| Race/ethnicity | In a large US registry study of over 304,000 patients with HNSCC diagnosed between 2000 and 2016, the racial distribution was 86.3% White, 10.6% Black, 2.0% Asian, and 0.9% Native American, with 3.9% identifying as Hispanic (3). SEER data for cancers of the oral cavity and pharynx, a major subset of HNSCC, show age-adjusted incidence rates per 100,000 of 20.9 in non-Hispanic White males, 12.8 in non-Hispanic Black males, 12.1 in non-Hispanic Asian/Pacific Islander males, and 10.5 in Hispanic males, with proportionally lower rates in females across all groups (4). |
| Geography | HNSCC is the sixth most common cancer globally, with an estimated 900,000 new cases and 500,000 deaths reported in 2020 (1, 2).  In Europe, approximately 90,000 new cases of HNSCC are diagnosed annually, with crude incidence rates of 4.6 per 100,000 for laryngeal, 3.5 per 100,000 for oral cavity, 3.3 per 100,000 for oropharyngeal, and 1.3 per 100,000 for hypopharyngeal cancers. Five-year relative survival was 61%, 49%, 41%, and 25% for laryngeal, oral cavity, oropharyngeal, and hypopharyngeal SCC, respectively (5).  India has a large head-and-neck cancer burden, with 225,419 new cases reported in 2020 and a 5-year prevalence of 480,944. Lip and oral cavity cancers accounted for most new cases (135,929) followed by larynx cancers (34,687). This etiologic profile is characterized by smokeless tobacco exposure. In India, per capita smokeless tobacco consumption was 18.4%, with 86.5% of people diagnosed with head-and-neck cancers reported to be tobacco users (6). |
| Other considerations | In the US, outcome disparities in HNSCC have been consistently documented across racial groups. Large registry analyses of over 304,000 patients show that 5-year OS is substantially lower in Black versus White patients (38.6% vs. 50.8%) (3). A multivariate analysis indicates that Black race is associated with a poorer OS vs. White race (HR 1.09, 95% CI 1.03–1.15; *p* = 0.002), with Black patients also more likely to present with advanced disease at diagnosis (3). Racial and socioeconomic factors, such as insurance status, education, and access to multidisciplinary care contribute to these disparities but do not fully account for them (3).  In Europe, race- or ethnicity-specific survival data are limited; however, multinational registry analyses show lower 5-year survival for head and neck cancers overall compared with Japan or Taiwan, suggesting geographic differences in disease outcomes and access to optimal care (7). |
| Overall representativeness of this study | The study population was broadly representative of patients with recurrent or metastatic HNSCC seen in clinical practice, with a median age (65 years) and male predominance (73%) consistent with epidemiologic trends. Most participants were White (69%), as expected for a trial conducted in European and US centers and aligning with US registry data (3); however, the assessment of outcomes across racial or ethnic groups is limited. The requirement for myCAF-rich tumors (≥5% CAFs) may select for a biologically distinct subset associated with poorer outcomes. Nevertheless, key demographic and clinical characteristics were comparable to those observed in another modern first-line HNSCC immunotherapy trial, KEYNOTE-048 (8), supporting the relevance of these findings to the broader HNSCC population with fibroblast-enriched disease. |

CI, confidence interval; HNSCC, head and neck squamous cell carcinoma; HPV, human papillomavirus; HR, hazard ratio; myCAF, myofibroblastic cancer–associated fibroblast; OS, overall survival; SCC, squamous cell carcinoma; SEER, Surveillance, Epidemiology, and End Results; US, United states.

**References**

1.	Bhatia A, Burtness B. Treating head and neck cancer in the age of immunotherapy: a 2023 update. Drugs 2023;83:217–48.

2.	Johnson DE, Burtness B, Leemans CR, Lui VWY, Bauman JE, Grandis JR. Head and neck squamous cell carcinoma. Nat Rev Dis Primers 2020;6:92.

3.	Baliga S, Yildiz VO, Bazan J, Palmer JD, Jhawar SR, Konieczkowski DJ, et al. Disparities in survival outcomes among racial/ethnic minorities with head and neck squamous cell cancer in the United States. Cancers (Basel) 2023;15:1781.

4.	National Cancer Institute Surveillance, Epidemiology, and End Results Program (SEER). Cancer Stat Facts: Oral Cavity and Pharynx Cancer 2026 [Available from: <https://seer.cancer.gov/statfacts/html/oralcav.html>.

5.	Machiels JP, Tao Y, Burtness B, Tahara M, Licitra L, Rischin D, et al. Pembrolizumab given concomitantly with chemoradiation and as maintenance therapy for locally advanced head and neck squamous cell carcinoma: KEYNOTE-412. Future Oncol 2020;16:1235–43.

6.	Noronha V, Prabhash K, Babu K G, Chaturvedi P, Kuriakose M, Birur P, et al. Introduction to Indian clinical practice consensus guidelines for the management of squamous cell carcinoma the head-and-neck: Update 2022. Cancer Res Stat Treat 2024;7:S1–5.

7.	Botta L, Matsuda T, Charvat H, Chiang CJ, Lee WC, van Gestel AJ, et al. Head and neck cancers survival in Europe, Taiwan, and Japan: results from RARECAREnet Asia based on a privacy-preserving federated infrastructure. Front Oncol 2023;13:1219111.

8.	Burtness B, Harrington KJ, Greil R, Soulières D, Tahara M, de Castro G, Jr., et al. Pembrolizumab alone or with chemotherapy versus cetuximab with chemotherapy for recurrent or metastatic squamous cell carcinoma of the head and neck (KEYNOTE-048): a randomised, open-label, phase 3 study. Lancet 2019;394:1915–28.
