# Supplementary material for: Setanaxib Added to Pembrolizumab for Fibroblast-Rich, Recurrent, or Metastatic Squamous Cell Carcinoma of the Head and Neck: A Phase II Randomized Study
Source: Cancer Res Commun. 2026 Oct 21;6(10):2463–74. doi: 10.1158/2767-9764.CRC-26-0422 (PMC13684902; doi:10.1158/2767-9764.CRC-26-0422)
Supplement: Supplementary file 9 [file crc-26-0422_supplementary_table_s2_suppst2.docx]

**Supplementary Table S2.** Prior, on-study, and subsequent therapies

|  | **Setanaxib 800 mg BID + pembrolizumab 200 mg Q3W**  **(*N* = 27)** | **Placebo + pembrolizumab 200 mg Q3W**  **(*N* = 28)** | **Overall**  **(*N* = 55)** |
| --- | --- | --- | --- |
| **Prior systemic anti-cancer therapy, n (%)** | | | |
| No prior therapy | 7 (25.9) | 10 (35.7) | 17 (30.9) |
| 1 prior therapy | 18 (66.7) | 18 (64.3) | 36 (65.5) |
| 2 prior therapies | 2 (7.4) | 0 | 2 (3.6) |
| **Prior radiation therapy, n (%)** | | | |
| No prior therapy | 2 (7.4) | 7 (25.0) | 9 (16.4) |
| 1 prior therapy | 14 (51.9) | 10 (35.7) | 24 (43.6) |
| 2 prior therapies | 8 (29.6) | 6 (21.4) | 14 (25.5) |
| 3 prior therapies | 3 (11.1) | 4 (14.3) | 7 (12.7) |
| 4 prior therapies | 0 | 0 | 0 |
| 5 prior therapies | 0 | 1 (3.6) | 1 (1.8) |
| **Prior cancer surgery, n (%)** | | | |
| No prior surgery | 14 (51.9) | 15 (53.6) | 29 (52.7) |
| Prior surgery | 13 (48.1) | 13 (46.4) | 26 (47.3) |
| **Most common concomitant medications during the study (>15% in the overall population), n (%)** | | | |
| Paracetamol | 15 (55.6) | 21 (75.0) | 36 (65.5) |
| Prednisolone | 10 (37.0) | 5 (17.9) | 15 (27.3) |
| Levothyroxine sodium | 8 (29.6) | 6 (21.4) | 14 (25.5) |
| Morphine sulfate | 7 (25.9) | 7 (25.0) | 14 (25.5) |
| Sodium bicarbonate | 5 (18.5) | 8 (28.6) | 13 (23.6) |
| Carboplatin | 7 (25.9) | 3 (10.7) | 10 (18.2) |
| Loperamide | 5 (18.5) | 5 (17.9) | 10 (18.2) |
| Macrogol 4000 | 3 (11.1) | 7 (25.0) | 10 (18.2) |
| Omeprazole | 3 (11.1) | 7 (25.0) | 10 (18.2) |
| Sodium chloride | 5 (18.5) | 5 (17.9) | 10 (18.2) |
| **Subsequent systemic anti-cancer therapy** | | | |
| No subsequent therapy | 15 (55.6) | 18 (64.3) | 33 (60.0) |
| Subsequent therapy | 12 (44.4) | 10 (35.7) | 22 (40.0) |
| **Subsequent radiation therapy, n (%)** | | | |
| No subsequent therapy | 27 (100) | 28 (100) | 55 (100) |
| Subsequent therapy | 0 | 0 | 0 |
| **On-study cancer surgery, n (%)** | | | |
| No on-study surgery | 25 (92.6) | 26 (92.9) | 51 (92.7) |
| On-study surgery | 2 (7.4) | 2 (7.1) | 4 (7.3) |

**Abbreviations:** BID, twice daily; Q3W, every 3 weeks.
