# Supplementary material for: Setanaxib Added to Pembrolizumab for Fibroblast-Rich, Recurrent, or Metastatic Squamous Cell Carcinoma of the Head and Neck: A Phase II Randomized Study
Source: Cancer Res Commun. 2026 Oct 21;6(10):2463–74. doi: 10.1158/2767-9764.CRC-26-0422 (PMC13684902; doi:10.1158/2767-9764.CRC-26-0422)
Supplement: Supplementary file 10 [file crc-26-0422_supplementary_table_s3_suppst3.docx]

**Supplementary Table S3.** Summary of progression-free survival

|  | **Setanaxib 800 mg BID + pembrolizumab  200 mg Q3W**  **(*N* = 27)** | **Placebo + pembrolizumab  200 mg Q3W**  **(*N* = 28)** |
| --- | --- | --- |
| **Status, n (%)** | | |
| Disease progression or death | 17 (63.0) | 21 (75.0) |
| Censored | 10 (37.0) | 7 (25.0) |
| Alive and progression free | 9 (33.3) | 5 (17.9) |
| Lacking disease evaluation after first study treatment | 1 (3.7) | 1 (3.6) |
| Premature study discontinuation/death after ≥2 consecutive missed tumor assessments | 0 | 1 (3.6) |
| **Progression-free survival percentile, days (80% CI)^a^** | | |
| 25% | 81 (63–125) | 60 (55–63) |
| 50% (median) | 151 (125–229) | 87 (65–117) |
| 75% | 315 (229–NC) | 184 (117–NC) |
| **Progression-free survival rate by timepoint (80% CI)^a^** | | |
| 3 months | 73 (62–84) | 48 (36–61) |
| 6 months | 41 (28–55) | 25 (14–37) |
| 12 months | 13 (0–27) | 0 |
| **HR^b^** | | |
| 80% CI | 0.58 (0.38–0.89) |  |
| p-value | 0.1023 |  |

Progression-free survival time (days) was calculated as [(date of disease progression or censoring) – (date of randomization)] + 1.

Patients who were not observed to have encountered the event were censored based on the rules detailed in the statistical analysis plan.

Overall visit responses were programmatically derived per RECIST 1.1, using the target response, non-target response, and new lesion indicator, collected on the Disease Response page of the eCRF for each visit.

^a^Obtained via Kaplan–Meier estimation (Brookmeyer–Crowley method).

^b^HR, CIs, and associated Wald chi-square p-value were obtained using a Cox proportional hazards model with treatment group as the independent variable.

**Abbreviations:** BID, twice daily; CI, confidence interval; eCRF, electronic case report form; HR, hazard ratio; Q3W, every 3 weeks; RECIST v1.1, Response Evaluation Criteria in Solid Tumors version 1.1.
