# Supplementary material for: Setanaxib Added to Pembrolizumab for Fibroblast-Rich, Recurrent, or Metastatic Squamous Cell Carcinoma of the Head and Neck: A Phase II Randomized Study
Source: Cancer Res Commun. 2026 Oct 21;6(10):2463–74. doi: 10.1158/2767-9764.CRC-26-0422 (PMC13684902; doi:10.1158/2767-9764.CRC-26-0422)
Supplement: Supplementary file 11 [file crc-26-0422_supplementary_table_s4_suppst4.docx]

**Supplementary Table S4.** Summary of overall survival

|  | **Setanaxib 800 mg BID + pembrolizumab 200 mg Q3W**  **(*N* = 27)** | **Placebo + pembrolizumab 200 mg  Q3W**  **(*N* = 28)** |
| --- | --- | --- |
| **Status, n (%)** | | |
| Death due to any cause | 6 (22.2) | 13 (46.4) |
| Censored | 21 (77.8) | 15 (53.6) |
| **Overall survival by timepoint (80% CI)** | | |
| 3 months | 96 (91–100) | 79 (69–88) |
| 6 months | 92 (86–99) | 68 (56–79) |
| 9 months | 88 (79–96) | 58 (46–71) |
| 12 months | 46 (19–72) | 42 (26–58) |
| **HR** | | |
| HR (80% CI) | 0.45 (0.24–0.85) | |
| p-value | 0.1057 | |

**Abbreviations:** BID, twice daily; CI, confidence interval; HR, hazard ratio; Q3W, every 3 weeks
