# Supplementary material for: Setanaxib Added to Pembrolizumab for Fibroblast-Rich, Recurrent, or Metastatic Squamous Cell Carcinoma of the Head and Neck: A Phase II Randomized Study
Source: Cancer Res Commun. 2026 Oct 21;6(10):2463–74. doi: 10.1158/2767-9764.CRC-26-0422 (PMC13684902; doi:10.1158/2767-9764.CRC-26-0422)
Supplement: Supplementary file 12 [file crc-26-0422_supplementary_table_s5_suppst5.docx]

**Supplementary Table S5.** Severe TEAEs (CTCAE grade ≥3) by system organ class (≥2 patients in either treatment group)

|  | **Setanaxib 800 mg BID + pembrolizumab  200 mg Q3W**  **(*N* = 27)** | **Placebo + pembrolizumab  200 mg Q3W**  **(*N* = 28)** | **Overall**  **(*N* = 55)** |
| --- | --- | --- | --- |
| **Any severe TEAE** | **11 (40.7)** | **14 (50.0)** | **25 (45.5)** |
| Respiratory, thoracic, or mediastinal disorders | 2 (7.4) | 5 (17.9) | 7 (12.7) |
| Blood or lymphatic system disorders | 3 (11.1) | 3 (10.7) | 6 (10.9) |
| Gastrointestinal disorders | 4 (14.8) | 2 (7.1) | 6 (10.9) |
| Infections and infestations | 2 (7.4) | 2 (7.1) | 4 (7.3) |
| Investigations^a^ | 4 (14.8) | 0 | 4 (7.3) |
| General disorders or administration site conditions | 2 (7.4) | 1 (3.6) | 3 (5.5) |
| Metabolism or nutrition disorders | 1 (3.7) | 2 (7.1) | 3 (5.5) |
| Neoplasms (benign, malignant, and unspecified, including cysts and polyps^b^) | 0 | 3 (10.7) | 3 (5.5) |
| Musculoskeletal or connective-tissue disorders | 0 | 2 (7.1) | 2 (3.6) |

Data are n (%).

^a^Lymphocyte count decreased, blood cholesterol increased, lipase increased, platelet count decreased.

^b^Tumor hemorrhage, tumor pain, tumor ulceration.

**Abbreviations:** BID, twice daily; CTCAE, Common Terminology Criteria for Adverse Events; Q3W, every 3 weeks; TEAE, treatment-emergent adverse event.
