# Supplementary material for: Setanaxib Added to Pembrolizumab for Fibroblast-Rich, Recurrent, or Metastatic Squamous Cell Carcinoma of the Head and Neck: A Phase II Randomized Study
Source: Cancer Res Commun. 2026 Oct 21;6(10):2463–74. doi: 10.1158/2767-9764.CRC-26-0422 (PMC13684902; doi:10.1158/2767-9764.CRC-26-0422)
Supplement: Supplementary file 13 [file crc-26-0422_supplementary_table_s6_suppst6.docx]

**Supplementary Table S6.** TEAEs leading to discontinuation of study drug by preferred term

|  | **Setanaxib 800 mg BID + pembrolizumab  200 mg Q3W**  **(*N* = 27)** | **Placebo + pembrolizumab  200 mg Q3W**  **(*N* = 28)** | **Overall**  **(*N* = 55)** |
| --- | --- | --- | --- |
| **Any TEAE leading to discontinuation of IMP** | **6 (22.2)** | **1 (3.6)** | **7 (12.7)** |
| Asthenia | 1 (3.7) | 1 (3.6) | 2 (3.6) |
| Colitis | 1 (3.7) | 0 | 1 (1.8) |
| Decreased appetite | 1 (3.7) | 0 | 1 (1.8) |
| Diarrhea | 1 (3.7) | 0 | 1 (1.8) |
| Hemoptysis | 1 (3.7) | 0 | 1 (1.8) |
| Headache | 1 (3.7) | 0 | 1 (1.8) |
| Lipase increased | 1 (3.7) | 0 | 1 (1.8) |
| Nausea | 0 | 1 (3.6) | 1 (1.8) |
| Pneumonia | 1 (3.7) | 0 | 1 (1.8) |
| Stomatitis | 1 (3.7) | 0 | 1 (1.8) |
| Weight decreased | 1 (3.7) | 0 | 1 (1.8) |

Data are n (%).

**Abbreviations:** BID, twice daily; IMP, investigational medicinal product; Q3W, every 3 weeks; TEAE, treatment-emergent adverse event.
