# Supplementary material for: Setanaxib Added to Pembrolizumab for Fibroblast-Rich, Recurrent, or Metastatic Squamous Cell Carcinoma of the Head and Neck: A Phase II Randomized Study
Source: Cancer Res Commun. 2026 Oct 21;6(10):2463–74. doi: 10.1158/2767-9764.CRC-26-0422 (PMC13684902; doi:10.1158/2767-9764.CRC-26-0422)
Supplement: Supplementary file 14 [file crc-26-0422_supplementary_figure_s4_suppsf4.docx]

**Supplementary Figure S4.** Kaplan–Meier plot for duration of response
